# Melt volume flow rate and melt flow rate of kenaf fibre reinforced Floreon/magnesium hydroxide biocomposites

**DOI:** 10.1186/s40064-016-3044-1

**Published:** 2016-09-29

**Authors:** C. H. Lee, S. M. Sapuan, J. H. Lee, M. R. Hassan

**Affiliations:** 1 Department of Mechanical Engineering, The University of Sheffield, Sheffield, S1 3JD UK; 2 Department of Mechanical and Manufacturing Engineering, Universiti Putra Malaysia (UPM), 43400 Serdang, Selangor Malaysia; 3 Laboratory of Biocomposite Technology, Institute of Tropical Forestry and Forest Products (INTROP), Universiti Putra Malaysia, 43400 Serdang, Selangor Malaysia; 4 The AMRC with Boeing, The University of Sheffield, Sheffield, S1 3JD UK

**Keywords:** Melt flow rate, Melt volume flow rate, Floreon, Kenaf fibre, Magnesium hydroxide, Biocomposites

## Abstract

A study of the melt volume flow rate (MVR) and the melt flow rate (MFR) of kenaf fibre (KF) reinforced Floreon (FLO) and magnesium hydroxide (MH) biocomposites under different temperatures (160–180 °C) and weight loadings (2.16, 5, 10 kg) is presented in this paper. FLO has the lowest values of MFR and MVR. The increment of the melt flow properties (MVR and MFR) has been found for KF or MH insertion due to the hydrolytic degradation of the polylactic acid in FLO. Deterioration of the entanglement density at high temperature, shear thinning and wall slip velocity were the possible causes for the higher melt flow properties. Increasing the KF loadings caused the higher melt flow properties while the higher MH contents created stronger bonding for higher macromolecular chain flow resistance, hence lower melt flow properties were recorded. However, the complicated melt flow behaviour of the KF reinforced FLO/MH biocomposites was found in this study. The high probability of KF–KF and KF–MH collisions was expected and there were more collisions for higher fibre and filler loading causing lower melt flow properties.

## Background

In recent years, natural fibre reinforcement with thermoset or thermoplastic polymer biocomposites have been studied intensively (Maleque et al. 2007; Sapuan and Harimi 2003; Sastra et al. 2006; Nur Aimi et al. 2014). Floreon (FLO) was developed by The University of Sheffield and CPD PLC in November 2013 (The Floreon Development Blog 2015). It is a biodegradable polymer which is constructed using standard polylactic acid (PLA). It was created for the greener, safer and better performance of the biopolymer. A lower manufacturing energy is required to produce FLO since it can be processed at about 160 °C, while most of the matrices require a temperature higher than 180 °C (Shukor et al. 2014; Ersoy and Taşdemir 2012; Liang et al. 2011; Libolon 2015; Lee et al. 2014). Besides this, it ensures a lower chance of fibre thermal degradation, especially for a low thermal stability natural fibre. FLO is a recyclable and fully biodegradable polymer. Mechanical recycling, as in the case of polyethylene terephthalate (PET), is applicable to FLO. This method requires less energy to reproduce recycled plastic (52.6 % less energy for recycling PET) as well as solving the landfill pollution problem (European Bioplastics 2015). On the other hand, feedstock recovery is an alternative option for FLO. This technique is currently applied to PLA and converting its product into the original material (lactic acid). A 99 % and above recovery rate has been claimed for PLA (Floreon 2015). Besides this, in-house testing has shown that FLO has better durability, strength and toughness. In addition, it has four times the impact resistance of PLA cast sheet specimens and almost twice the toughness of PET (Duc et al. 2011).

Melt flow properties have provided a significant insight for polymer manufacturing. MVR and MFR are indicators of the flow properties of the material in melt. Investigations have been performed with respect to the MVR of PLA composites with different temperatures and loadings (Nur Aimi et al. 2014). A higher MVR value was found with increased temperature due to the increase of the melt free volume. A higher applied load also resulted in a higher MVR due to the shear thinning effect. On the other hand, lower MFR properties of PLA compared to polypropylene (PP) have been found, e.g. poorer wetting on KF, leading to weak fibre-polymer interaction and causing lower strength properties (Han et al. 2012). Also, it has been reported that the viscosity increases with the loading of KF in composites; this is because changes in molecular weight are caused by KF and the interaction between the fibres and the matrix (Mohammad and Arsad 2013). Another study has been conducted which concerns the effect of MH particle size on the PP matrix (Yang et al. 2009). Decreasing melt flow properties were shown for the composites for particle sizes up to 5 µm, yet an increase in melt flow properties was found for MH particle sizes larger than 5 µm. This is because the small particles enhanced the macromolecular chain flow resistance, while the larger particle sizes reduced the flow resistance as a decreased distance was found between the flame retardant particles (Yang et al. 2009).

From the above reviews, it is evident that no previous work has been conducted on the MFR of the KF reinforced FLO biocomposite with MH used as a flame retardant filler. Therefore, the aim of the present work is to study MH inclusion and the MVR and MFR of KF reinforced FLO biocomposites.

## Experimental section

### Materials

**Fig. 1:**
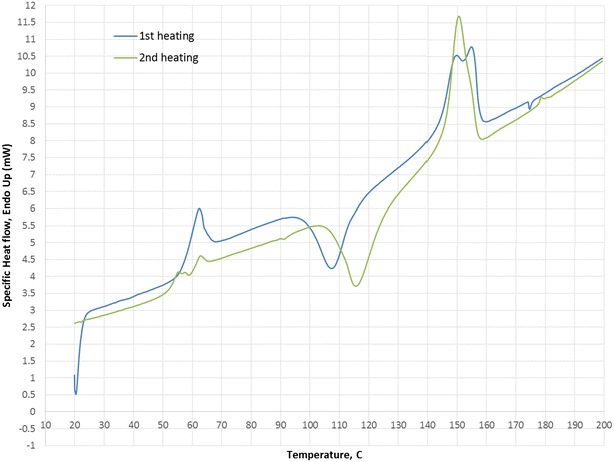
DSC curve of the FLO polymer

The FLO biopolymer Grade 100 was contributed by The University of Sheffield and was used as the matrix. As shown in Fig. 1, the differential scanning calorimetry (DSC) curves of previous work indicate the melting peak temperature for the first and second heating cycle. The first heating cycle was intended to remove the thermal history of the polymer. The melting temperature is 150.5 °C. KF with average length of 8–15 mm was obtained from Tazdiq Engineering, Serdang, Malaysia, in order to reinforce the composites. MH was supplied by Fisher Scientific UK Ltd with 95 % purity and was used as a non-toxic flame retardant to enhance the material’s fire barrier properties. The sodium hydroxide used in the alkaline treatment was supplied by APC Pure, UK.

### Processing methods

Nine samples with different ratios of FLO biocomposite were prepared using a 21 mm lab twin screw extruder (Table 1). KF was initially dried at 50 °C for 24 h before undergoing 6 % NaOH treatment for 4 h. Then the KF was washed with water and dried at 100 °C for 6 h. All combinations of the composite were simply blended by hand. The extrusion (L/D = 30) was performed at 50 RPM and 180 °C at the die head and increasing to 186 °C at the feed section. The extruded strands were then air-dried and pelletised. The pellets were then tested for their melt flow properties.

**Table 1:** Composition of the FLO biocomposites

Sample	Floreon (wt%)	Kenaf, fibre (wt%)	Magnesium hydroxide (wt%)
1	100	–	–
2	95	5	–
3	90	10	–
4	95	–	5
5	90	–	10
6	90	5	5
7	85	5	10
8	85	10	5
9	80	10	10

### Characterisation

#### Melt flow index testing

The main experimental instrument used in this work was the Mflow extrusion plastometer, which was supplied by Zwick Testing Machines Ltd. The machine was located in a laboratory of The University of Sheffield, UK. The melt flow properties of the composites were measured in the temperature range 160–180 °C and for weight loadings of 2.16, 5 and 10 kg. The weight of the die rod was 0.325 kg and the die diameter was 8.26 mm. 300 s of pre-heating was conducted after a measured amount of sample was put into the machine’s chamber.

## Results section

**Fig. 2:**
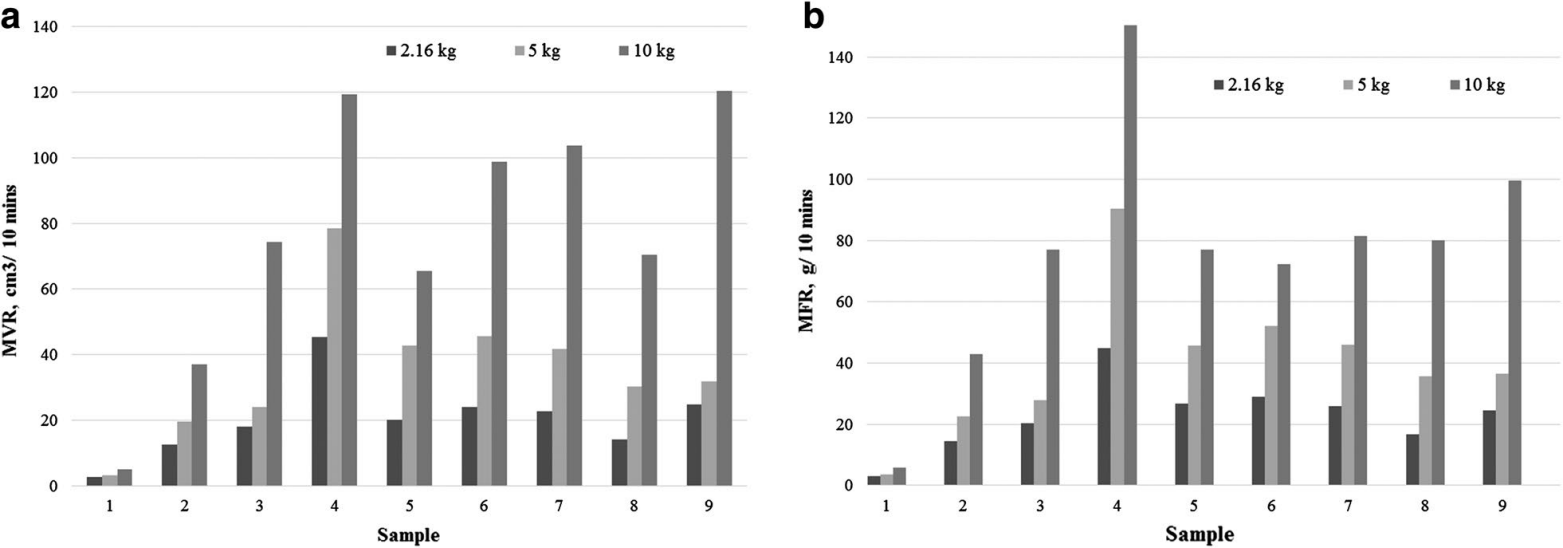
**a** MVR, **b** MFR of the FLO biocomposites under different loading at 170 °C

Figure 2a, b shows the MVR and the MFR of the FLO biocomposites under different loadings at 170 °C. Both the indices (MVR and MFR) increased with the loading applied. Under a high loading, a high shear rate is exerted at the wall along the channel. A common phenomenon known as “shear thinning” exists and this effect is found to be more obvious at higher weight loading. Shear thinning is an effect whereby there is a higher flow rate for increasing shear rate under constant temperature (Liang et al. 2011).

Alkaline treated KF has largely decreased the amount of impurities and hence given a smoother surface to the fibres in the composites. A ball-bearing effect has been performed by the KF and this has resulted in a higher MFR (Liang et al. 1999). When the KF with a random arrangement is applied to the shear, the fibres are forced to align in the flow direction (shear direction). The degree of alignment depends on the shear rates, and a high shear rate causes almost complete alignment and thus higher flow rates (Lafranche et al. 2015). Besides this, a large portion of the flow velocity was contributed by the wall slip especially for low weight loading. The change in the wall slip velocity is greatly influenced by the fibre content in the composites, rather than merely by the polymer behaviour at the die surface. A previous study showed a 100 % wall slip contribution at a low shear level for 60 % maple HDPE composites and 40 % pine composites (Li and Wolcott 2004). More work needs to be done to determine how much flow velocity was contributed by the wall slip theory.

Ersoy and Taşdemir (2012) indicated a more than 52.22 % decline of MFR from 0 to 20 wt% of MH filled composites. By increasing the MH content from 5 to 10 wt% (sample 4–5), the MH particles formed new network junctions in the composites, resulting in better interaction forces and friction forces (Crowson et al. 1980; Khalina et al. 2011). Therefore lower MVR and MFR values for the composites were found with increasing MH contents (Liang et al. 2000).

**Fig. 3:**
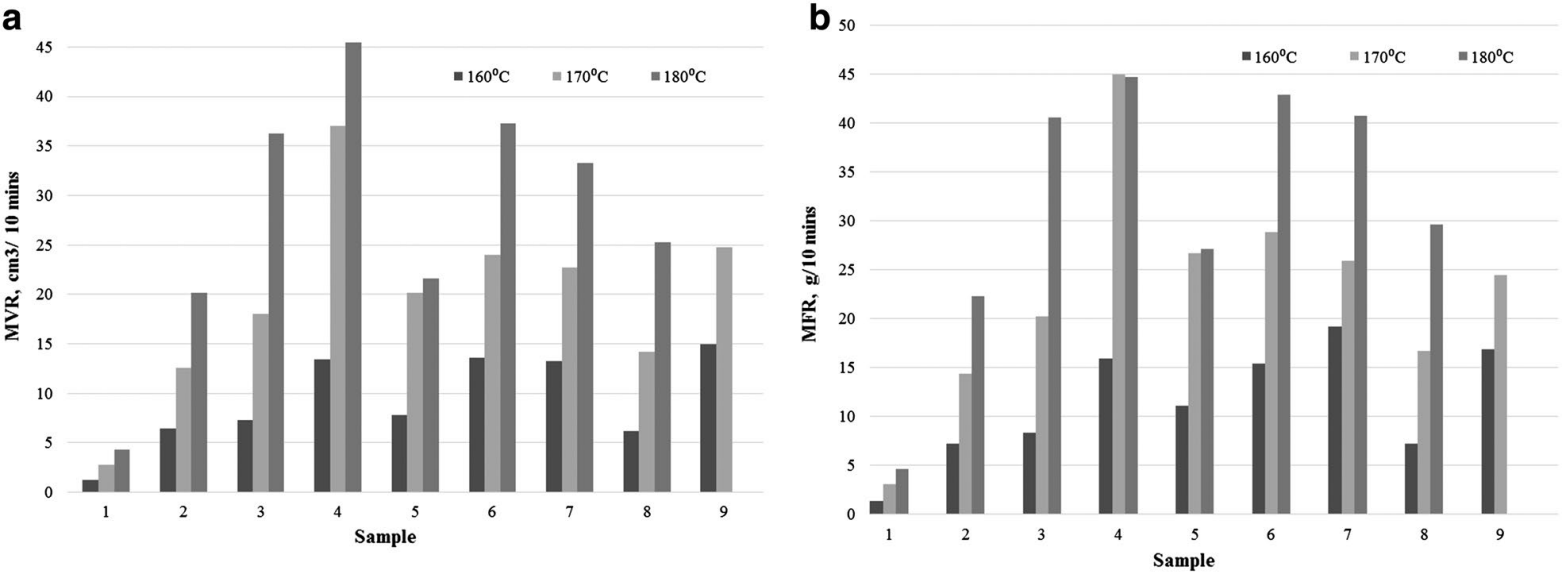
**a** MVR, **b** MFR of the FLO biocomposites under a constant load of 2.16 kg across the temperature range from 160 to 180 °C

Figure 3a, b show the MVR and the MFR of the FLO biocomposites under a constant load of 2.16 kg across the temperature range of 160–180 °C. It can be seen from Fig. 3a, b that both the MVR and the MFR were found to increase with temperature. The polymer molecules absorbed the heat energy and weakened at high temperature. The weakened polymer has a higher free volume in specific weight and hence this resulted in a higher MVR. On the other hand, the higher temperature led to the deterioration of the entanglement density. This caused the sample to flow faster and higher in the MFR since the molecular layers became more slippery (Liang et al. 1999; Lafranche et al. 2015). There was also an increment in the activation energy for the polymer molecules at higher temperature. Hence, an increase in the MFR was found, which agreed with previous work (Gilbert et al. 1982). On the other hand, sample 9 was unable to undergo the test after the pre-heating stage at 180 °C. The sample expanded significantly in the chamber while pre-heating, restricting it from flowing.

**Fig. 4:**
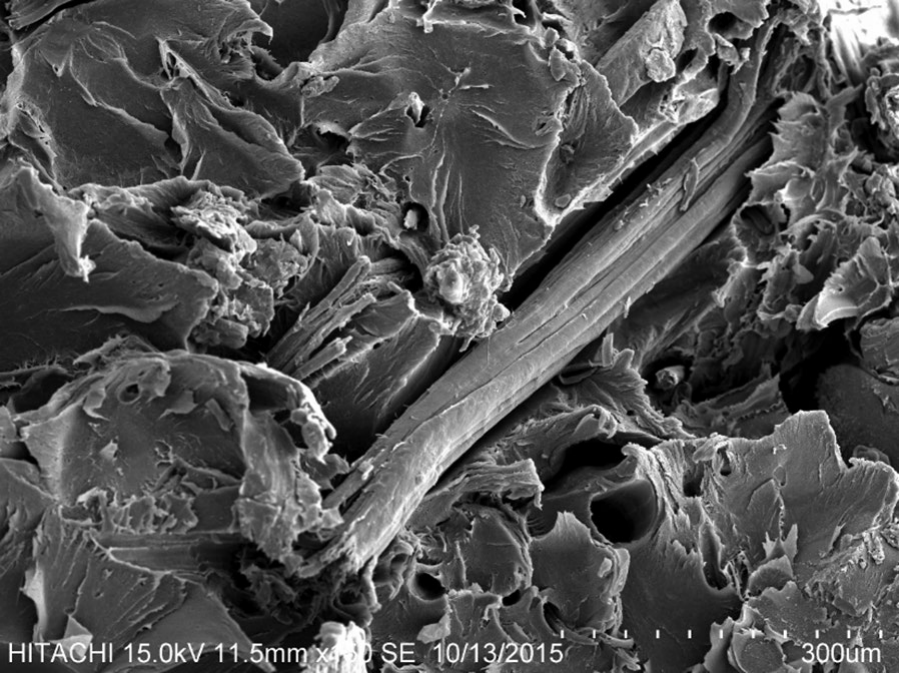
Scanning electron micrograph of sample 3

FLO has the lowest flow index values compared to its composites. It is believed that the insertion of the KF and MH disturbed the molecular chain of the FLO. The hydrophilic nature of KF and MH induced the hydrolytic degradation of the PLA in FLO (Li 1999; Tsuji and Ikarashi 2004; Baimark and Srihanam 2015). This reduced the polymer’s molecular length, and flow is easier with a shorter length (Gorrasi and Pantani 2013; Liang and Peng 2009). At the same time, a poor interaction between KF and FLO was found in sample 3 using a scanning electron micrograph (Fig. 4); they were expected to have a higher flow capability. On the other hand, the insertion of MH (5 wt%) caused a significant increase in the melt flow properties. However, further MH insertion (10 wt%) created new bonding in the composites (Ersoy and Taşdemir 2012). Therefore the drop in the values for the melt flow properties indicated that strong bonding has resisted the flow. The complicated melt flow behaviour of KF reinforced FLO/MH biocomposites has been found in this study. The addition of KF to the biocomposites (samples 6–7) has been found to increase the MFR at 160 °C but decreased the MFR for temperatures of 170 and 180 °C. KF is disoriented in composites and the MH disturbs the converging flow in the die entrance due to the natural fibre reinforced polymer composites. Therefore a high probability of KF–KF/KF–MH collisions is expected and there are more collisions with higher fibre loading, which lowers the MFR (Liang et al. 2010). On the other hand, increasing the MH loading in the MH biocomposites has constantly decreased the MFR and MVR values, showing a stronger bonding in the biocomposites.

## Conclusions

The melt flow properties of the KF reinforced FLO/MH bicomposites have been studied for varying temperatures and weight loads. In general, the melt flow capability has been increased for higher temperatures and weight loads. This is because the macromolecular chain has absorbed more heat at higher temperature, causing a weaker bonding. On the other hand, the shear thinning effect has been found for higher weight loadings, resulting in higher melt flow properties. FLO has the lowest melt flow indices; it is believed that the hydroxyl groups from the KF and the MH induced hydrolytic degradation on the PLA in FLO. A high content of MH induced new network junctions with better interaction forces and friction forces, causing lower MFR and MVR values. On the other hand, the smooth surface of KF has been forced to align in the flow direction when a load is applied, resulting in higher melt flow properties. Besides this, a large portion of the flow velocity was contributed by the wall slip theory, especially for low weight loading. However, complicated melt flow behaviour for KF reinforced FLO/MH biocomposites has been found in this study. A high probability of KF–KF/KF–MH collisions is expected and there are more collisions with higher fibre loading. On the other hand, increasing the MH loading in the MH biocomposites has constantly decreased the MFR and MVR values, showing that the stronger bonding in the biocomposites has resisted the macromolecular chain flow.

## Abbreviations

MVR: melt volume flow rate
MFR: melt flow rate
KF: kenaf fibres
FLO: Floreon
MH: magnesium hydroxide
PLA: polylactic acid
PET: polyethylene terephthalate
